# Risk of Parkinson's disease after tamoxifen treatment

**DOI:** 10.1186/1471-2377-10-23

**Published:** 2010-04-12

**Authors:** Jeanne C Latourelle, Merete Dybdahl, Anita L Destefano, Richard H Myers, Timothy L Lash

**Affiliations:** 1 Department of Neurology, Boston University School of Medicine, 85 East Concord Street, Boston MA, 02118, USA; 2 Department of Epidemiology Boston University School of School of Public Health, 85 East Concord Street, Boston MA, 02118, USA; 3 Department of Clinical Epidemiology, Aarhus University, Nordre Ringgade 1, DK-8000, Aarhus, Denmark; 4 Department of Biostatistics, Boston University School of School of Public Health, 85 East Concord Street, Boston MA, 02118, USA

## Abstract

**Background:**

Women have a reduced risk of developing Parkinson's disease (PD) compared with age-matched men. Neuro-protective effects of estrogen potentially explain this difference. Tamoxifen, commonly used in breast cancer treatment, may interfere with the protective effects of estrogen and increase risk of PD. We compared the rate of PD in Danish breast cancer patients treated with tamoxifen to the rate among those not treated with tamoxifen.

**Methods:**

A cohort of 15,419 breast cancer patients identified from the Danish Breast Cancer Collaborative Group database was linked to the National Registry of Patients to identify PD diagnoses. Overall risk and rate of PD following identification into the study was compared between patients treated with tamoxifen as adjuvant hormonal therapy and patients not receiving tamoxifen. Time-dependent effects of tamoxifen treatment on PD rate were examined to estimate the likely induction period for tamoxifen.

**Results:**

In total, 35 cases of PD were identified among the 15,419 breast cancer patients. No overall effect of tamoxifen on rate of PD was observed (HR = 1.3, 95% CI: 0.64-2.5), but a PD hazard ratio of 5.1 (95% CI: 1.0-25) was seen four to six years following initiation of tamoxifen treatment.

**Conclusions:**

These results provide evidence that the neuro-protective properties of estrogen against PD occurrence may be disrupted by tamoxifen therapy. Tamoxifen treatments may be associated with an increased rate of PD; however these effects act after four years, are of limited duration, and the adverse effect is overwhelmed by the protection against breast recurrence conferred by tamoxifen therapy.

## Background

Parkinson's disease (PD) is a degenerative movement disorder usually occurring late in life. It is the second most common neurodegenerative disease after Alzheimer disease (AD) and has a prevalence of approximately 1.8% among people over the age of 65 [1,2]. Women have a reduced risk of Parkinson's disease compared to age-matched men [1]. This rate difference, combined with evidence of neuro-protective properties of estrogen in AD, and probably also in other neurodegenerative disorders [3], has prompted studies of estrogen's effect on PD risk and treatment. Improved motor function has been associated with estrogen treatment in studies of women with PD [4-6]. Furthermore, studies have shown some factors associated with estrogen, such as hormone replacement therapy [7], length of fertile life [8], or receipt of hysterectomy [9] are also associated with PD risk. Other studies however, have shown no or an inverse association with the same or similar factors [8-12]. Selective estrogen receptor modulators (SERMs) are a class of pharmaceuticals that can act as both estrogens and anti-estrogens in different tissues [13]. Tamoxifen was the first SERM developed, and the observed anti-estrogenic properties led to its use in treatment of breast cancer [13,14]. Tamoxifen decreases mortality and recurrence rates in breast cancer patients, and prevents breast cancer occurrence in high-risk women [15,16]. Tamoxifen is most effective in treating and preventing the approximately 70% of breast cancer tumors that express *ESR1 *(the gene encoding the Estrogen Receptor-α protein), classified as estrogen receptor positive (ER+) tumors [15,17].

The mechanisms by which SERMs act are not completely clear, however it is known that SERMs bind to the estrogen receptors (ERs) and form ER complexes similar to the estrogen receptor-estrogens complexes. These complexes can then either promote gene transcription, similar to estrogen, become misfolded and prevent gene transcription, or become misfolded and promote a different set of gene transcription [13,14].

As tamoxifen has become widely used for treatment and prevention of breast cancer [15,18], interest in the effects of SERMs on the brain has grown. It is not clear whether tamoxifen acts as an estrogen or anti-estrogen in the brain, although animal studies of serotonin transport suggest that SERMs can act as an antagonist to estrogen in at least some parts of the brain [19,20]. Studies of the relation between SERM treatment and PD have mainly focused on animal models of PD induction, in particular PD induced by 1-methyl-4-phenyl-1,2,3,6-tetrahydropyridine (MPTP) or methamphetamine (MA) in mouse models.

In these studies, estrogen decreases the toxic effects of MPTP in male and female mice, but simultaneous administration of tamoxifen blocks the protective effect of estrogen against MPTP induced PD [21-24]. In addition, MPTP-treated and MA-treated mice, when administered tamoxifen and estrogen together, have significantly reduced dopamine levels compared with mice administered estrogen alone. This result suggests an anti-estrogenic effect of tamoxifen on the dopaminergic neurons affected in PD [24,25]. These animal studies indicate that tamoxifen may oppose the neuro-protective benefits of estrogen in the brain, particularly in the parts of the brain affected by PD. Nevertheless, no epidemiologic studies of the association between tamoxifen treatment and PD occurrence have been conducted.

In this study, we have identified a cohort of breast cancer patients registered by the Danish Breast Cancer Collaborative Group (DBCG) database and linked the cohort to the National Registry of Patients covering all Danish hospitals. We compared the rates of Parkinson's disease diagnosis between those patients whose treatment included tamoxifen and those whose treatment did not include tamoxifen.

## Methods

### Study Population

An initial cohort of 15,440 women between the ages of 45 and 70 years, diagnosed with stage I or II, estrogen receptor positive (ER+) breast cancer between 1990 and 2004 were identified from the Danish Breast Cancer Collaborative Group (DBCG). The DBCG was created in 1977 to ensure comprehensive diagnosis, treatment, and study of breast cancer in Denmark [26] and maintains detailed information about nationwide breast cancer diagnoses, outcomes, and treatments, including tamoxifen treatment. The Danish national health care system enables linkage of the breast cancer patient cohorts to the Danish National Registry of Patients to ascertain subsequent PD diagnosis. Tamoxifen treatment is only indicated in ER+ breast cancer and few ER- cases are treated with tamoxifen. A relationship between ER status and risk of PD, possibly mediated by cumulative estrogen exposure [27], may also exist. Therefore to avoid confounding by ER status, only women with ER+ breast cancer were included in this study.

Use of tamoxifen was determined from the assigned treatment protocol for each patient collected from the DBCG database [28]. Tamoxifen exposure was defined for this study by the assigned treatment protocol for each patient to reduce confounding by non-compliance and other factors. Determination of treatment protocols was dependent on the risk profile of the patient, which was determined mainly by the cancer stage. Patients defined as low-risk (generally stage I patients, with smaller tumors that had not metastasized locally) were not systematically assigned therapy through the DBCG, but were treated at the discretion of their physicians [29]. High-risk patients included in this study (stage II with larger tumors or tumors that had metastasized locally) were recommended standardized treatment based on menopausal status and estrogen receptor status. In addition, some patients in the high-risk group were randomized to one of several clinical trials [29]. Because the women with stage II cancer were subject to standardized treatment protocols, there is less potential for confounding by unmeasured variables (such as smoking) in this group.

Follow-up time was measured as the interval from the date of breast cancer surgery, when treatment protocols were assigned, to the date of first diagnosis of PD or to censoring due to death, emigration, or the end of the study period (12/31/2005). To reduce misclassification of the PD outcome, follow-up time was also censored at the time of a diagnosis of brain metastasis, because symptoms of brain metastasis could be misinterpreted as PD onset. To identify the date of initial hospital diagnosis of PD, the Central Population Register (CPR) identification number assigned to all Danish citizens was used to query the Danish National Registry of Patients (NRP). The NRP records all outpatient visits after 1994 and all hospital admissions after 1977 and includes the date of the specialist visit or hospital discharge, and codes (based on International Classification of Disease version 8 or 10 codes) for up to 20 different disease diagnoses identified as related to the visit by the health care provider [30]. The date of initial diagnosis of PD is therefore the first date of any visit or discharge associated with one of the International Classification of Disease codes for PD (ICD8 342.0 or ICD10 G20[31]) in the National Registry of Patients. Eighteen women with PD diagnostic codes identified before breast cancer diagnosis were excluded from the study. In addition, two patients diagnosed with both PD and brain cancer and one patient diagnosed with brain cancer before breast cancer diagnosis were excluded from the study to avoid potential misclassification of the PD outcome. These exclusions reduced the initial cohort of 15,440 to a final cohort of 15,419 patients.

This study was approved by the Danish Registry Board. There is no requirement for registry research of this sort to be further approved by an ethics board in Denmark.

### Statistical Analyses

We computed the crude incidence rate of PD for women from the study cohort assigned to tamoxifen treatment (N = 7153) and compared it with the rate among women from the study cohort who were not assigned tamoxifen treatment (N = 8266). We used proportional hazard regression implemented in SAS version 9 to calculate a hazard ratio associating tamoxifen exposure with PD incidence. Potential confounding by age of breast cancer diagnosis, date of treatment assignment, and breast cancer stage was assessed by determining the association between potential confounders and the disease in the unexposed group (tamoxifen untreated), and by comparing the change in estimates of the association between tamoxifen and PD occurrence when controlling for potential confounding variables. Age and calendar time of cancer diagnosis and treatment were found to be associated with, and were found to modify, the effect of tamoxifen treatment rate of PD. The hazard ratio was therefore also calculated adjusting for age at breast cancer diagnosis and date of treatment assignment. In addition, analyses were performed in only high-risk patients (stage II), who were subject to standardized treatment protocols, to reduce confounding by unmeasured variables (such as smoking).

Because the potential induction time between tamoxifen exposure and onset of PD is uncertain, PD-free survival curves and log-log survival plots were generated using the Lifetest procedure in SAS to examine the validity of the proportional hazards assumption. We expected that there would be an induction time immediately following the start of tamoxifen treatment, during which the drug would not have had an opportunity to have a pharmacologic effect on PD occurrence. Similarly, we expected that there would be a point in time after treatment was completed by which it would no longer affect PD risk. To assess these induction period effects on PD rate, we repeated the proportional hazards regression within strata of the estimated periods before, during, and after which the tamoxifen treatment may have had an effect on PD occurrence. The boundaries of these periods were determined empirically by examination of the PD-free survival curves and log-log survival plots.

## Results

In the final cohort of 15,419 ER+ breast cancer patients, 7153 patients were assigned to tamoxifen treatment protocols and 8266 were not. Descriptive statistics including age, cancer stage, and assigned tamoxifen treatment duration are described in Table 1. In total, 35 cases of PD were identified; 15 in the tamoxifen treated cohort and 20 in the cohort not treated with tamoxifen. The unadjusted hazard ratio was 1.7 (95% CI of 0.83-3.3). Adjustment for age at breast cancer diagnosis and date of treatment assignment reduced the hazard ratio to 1.3 (95% CI 0.64-2.5). Analyses restricted to advanced stage cases, thus excluding stage I cases who were not subject to systematic treatment recommendations, showed a similar association, with an age and date adjusted hazard ratio of 1.3 (95% CI 0.47-3.5).

**Table 1:** The age distribution, cancer stage, and assigned treatment protocols for 15,419 female ER+ breast cancer patients

		All	Tamoxifen exposed	Tamoxifen unexposed
All ER+ women		15419	7153	8266

Age-range				
	45-50	3377	1129	2248
	51-55	3127	1536	1591
	56-60	3317	1695	1622
	61-65	3229	1649	1580
	66-70	2369	1144	1225

Stage of cancer				
	1	6823	907	5916
	2	8596	6246	2350

Assigned tamoxifen treatment duration				
	180 days	354	354	
	365 days	990	990	
	730 days	620	620	
	1825 days	5039	5039	
	none	8266		8266
	*missing*	*150*	*150*	

PD cases identified		35	15	20

Incidence Rate (cases/10,000 person-years)		3.38	4.05	3.01

**Figure 1:**
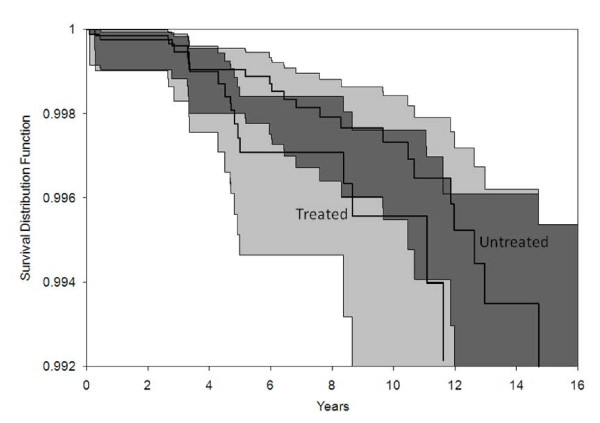
**PD-Free Survival Curves**. The PD-free Survival curves and confidence intervals for tamoxifen treated and untreated are shown. Dark grey shading indicates where the confidence intervals overlap.

Figure 1 shows the PD-free survival curves with confidence intervals for the tamoxifen treated and untreated groups. The confidence intervals overlap at all time periods (shown by the dark grey shading), indicating no significant overall difference in survival between the groups. Nevertheless, the shapes of the curves indicate a violation of the proportional hazards assumption approximately four to six years after breast cancer diagnosis (approximately the time of tamoxifen inception). Instead of displaying the steadily increasing gap expected under proportional hazards, the survival curves start close to one another, diverge quickly between years four and six, and then maintain a near uniform distance. This pattern indicates a time-dependent effect of tamoxifen treatment on PD occurrence.

The results of three independent proportional hazard regression models representing the periods before, during, and after this presumed window of effect of tamoxifen treatment on PD are shown in Table 2. The period four to six years after beginning treatment is associated with a hazard ratio of 5.1 (95% CI 1.02 to 25), while exposure before and after that range shows little effect on PD hazard.

**Table 2:** Hazard ratios for effect of tamoxifen in three time ranges after exposure

Time from exposure	N Cases Observed (N Cases exposed)	Hazard Ratio	Lower 95% CI	Upper 95% CI	p-value
0-4 years	11 (5)	0.89	0.26	3.0	0.84

4-6 years	8 (6)	5.1	1.02	25	0.047

6+ years	16 (4)	0.79	0.25	2. 5	0.68

## Discussion

An examination of the effect of tamoxifen treatment on PD rate among 15,419 female breast cancer cases in the Danish Breast Cancer Collaborative group did not show a significant difference in rate of PD overall. Our results do, however, suggest a time-dependent effect starting around four years after inception of tamoxifen therapy, which was born out in stratified hazard analyses. Estimated hazard ratios over the first three years after beginning tamoxifen treatment and more than six years after beginning treatment showed little or no effect of tamoxifen on the rate of PD. In the range of 4 to 6 years, however, an increase in the hazard ratio was observed. This pattern of time-varying effect is consistent with the theory that certain exposures will only show an effect within a limited time frame, long enough after exposure for the effect to become apparent, but which ceases when the exposure is no longer active. While the evidence for an effect of tamoxifen treatment on PD risk is equivocal, this effect suggests that treatment may hasten the occurrence of PD pathology or symptoms. This result is also consistent with the hypothesis and animal-model evidence that estrogen has a neuro-protective effect that functions through a mechanism that can be disrupted by tamoxifen, likely through estrogen receptors.

The animal-model studies have not investigated time-dependent effects of tamoxifen in the brain. Clinical trials of differing durations of tamoxifen treatment for breast cancer show that a 5 year duration of tamoxifen treatment is more effective than shorter treatments [32], consistent with the view that tamoxifen treatment does not induce a permanent biological effect, but rather a time-limited effect that eventually wanes. Interestingly, a study of potential carryover effects of tamoxifen in the Early Breast Cancer Trialists' Collaborative Group (EBCTCG) suggests that the majority of tamoxifen's benefit in reducing recurrence risk occurred within the first five years after initiating treatment and no additional benefit was detected after six years [33], consistent with the endpoint of effect on hazard of PD in this study. The induction period of four years seen in this study is not consistent with the effects of tamoxifen seen in breast cancer trials, though the pre-clinical progression and latency period of PD is not well understood [34], making it difficult to speculate on an appropriate induction period.

The major strength of this study is that the DBCG and the Danish National Registry of Patients provide a large well-characterized nationwide cohort of breast cancer cases for study. Such a large cohort of tamoxifen-treated breast cancer patients with the ability to assess neurological outcomes is very rare. Despite the large cohort, however, only a limited number of PD cases were identified. The low frequency is due to the rarity of PD in general. The age-specific incidence rates of PD in these women (15.6/100,000 person-years for ages 50-64, 58.6/100,000 person-years for ages 65-79) are similar to the rates in the general population of women in Denmark (9.4/100,000 person-years for ages 50-64, 57.6/100,000 person-years for ages 65-79)[31]A second limitation is the possibility for uncontrolled confounding by smoking history or caffeine use. Former smoking has been consistently shown to reduce the rate of PD occurrence [35,36], and caffeine use may also be associated with a decreased risk of the disease particularly in women [11,12,37], but neither smoking nor caffeine information was available for members of this cohort. Cigarette smoking is common in women in Denmark, with a prevalence of 45% in women age 35 to 64 compared with a median of 24% in other European countries [38,39]. Smoking is at most a weak risk factor for breast cancer, but as the study only included breast cancer patients, of greater concern is whether smoking may be associated with receipt of tamoxifen therapy among breast cancer patient. (In personal communications, Danish oncologists report that smoking status would not be a factor considered in prescribing tamoxifen treatment for breast cancer.) Nevertheless, studies have shown an increased risk of tamoxifen related side effects in smokers [40] and it may be possible for some physicians concerned about tamoxifen related side effects to be less likely to prescribe tamoxifen treatment to smokers. If more non-smokers, at higher risk of PD, were exposed to tamoxifen, uncontrolled confounding could potentially influence the results seen in this study. It is unlikely that this confounding would have a strong effect only in the range of four to six years after initiation of tamoxifen therapy, during which the strongest effect was seen in this study, but it could influence the overall increased rate of PD seen in the tamoxifen exposed group.

To assess the potential bias due to uncontrolled confounding by smoking, we conducted a quantitative bias analysis [41]. We used a previously estimated relative rate of PD of 0.7 comparing smokers with nonsmokers [42] and an estimated OR of 1.5 for the association between smoking and tamoxifen treatment. This OR assumes smokers were two-thirds as likely to receive tamoxifen treatment as non-smokers, all other factors held constant. With these assumptions, the adjusted hazard ratio of PD associated with tamoxifen treatment would decrease only slightly from 1.7 to 1.3. Even with an implausibly strong association between smoking and receipt of tamoxifen treatment (OR = 5), external adjustment for smoking does not completely remove the positive association between PD and tamoxifen (predicted RR of 1.20).

A third limitation is the potential for some misclassification of PD and time of PD diagnosis ascertained through the codes in the Danish National Registry of Patients. A validation study conducted within the Registry of Patients compared diagnoses in medical records to the registry codes and estimated a false-positive rate of PD diagnosis between 6% and 20% [43] in the registry. No examination of the false-negative rate was performed in this study, but as PD is a rare disease, virtually all people classified as non-PD are likely to be truly non-PD. The criteria used for defining PD from the medical records in the validation study was not specifically explained, so it is uncertain if this validation corresponds to commonly-used criteria (such as the United Kingdom Brain Bank criteria [44]). A study of the purchase of anti-parkinsonism drugs (APD) using the Danish Medicinal Product Statistics database suggests that the incident rate of APD purchases is twice as high (43.4/100,000 person-years) [45] as the incident rate of PD (19.9/100,000 person-years) estimated from the National Registry of Patients [31]. This study did not match APD drug purchase to disease diagnoses or drug indication, thus the discrepancy may be due to the use of APD for clinical purposes other than PD treatment, but may also indicate an underreporting of PD in the National Registry of Patients [45]. It is unlikely that misclassification of PD status would occur at different rates between breast cancer patients treated or not treated with tamoxifen, and thus we expect PD misclassification would bias the estimated associations toward the null.

## Conclusions

These findings are based on a very small number of PD cases, as PD remains a rare disease, particularly among women. In addition, the determination of the time-frame of potential effect was determined empirically from study data. Only six exposed cases of PD were diagnosed during the period of potential effect four to six years after breast cancer diagnosis. While this frequency is greater than expected, caution should be used in interpreting these results due to the small number of observed cases and empirical delineation of the induction period. Ideally, these findings would spur efforts at replication in another cohort. It is also important to remember that while the hazard ratio of 5.1 represents a large relative effect, the absolute increased rate of PD is negligible when compared to the benefits of tamoxifen in preventing breast cancer recurrences. The main value of this study's result, therefore, is its contribution to understanding a potential underlying disease mechanism in PD and particularly the effect of estrogen on the disease process.

## Competing interests

The authors declare that they have no competing interests.

## Authors' contributions

JCL participated in the design of the study, conducted statistical analyses, participated in the interpretation of data, and drafted the manuscript. MD participated in the design of the study, performed data collection and cleaning, and revised the article critically for important intellectual content. ALD and RHM participated in the interpretation of data, and revised the article critically for important intellectual content. TLL participated in the design and coordination of the study, participated in the interpretation of data, and revised the article critically for important intellectual content. All authors read and approved the final manuscript.

## Pre-publication history

The pre-publication history for this paper can be accessed here:

http://www.biomedcentral.com/1471-2377/10/23/prepub
